# Socioeconomic Status and Long-Term Care Provision to Parents in Japan

**DOI:** 10.1093/geroni/igaa057.2568

**Published:** 2020-12-16

**Authors:** Yoko Ibuka, Yui Ohtsu

**Affiliations:** 1 Keio University, Tokyo, Japan; 2 National Institute of Population and Social Security Research, Tokyo, Tokyo, Japan

## Abstract

Socioeconomic status (SES) is generating considerable interest in terms of health of individuals, but how it is associated with long-term care has not been established yet. We study the relationship between SES and long-term care provision to parents among the Japanese adults using JSTAR. We use the following six measures of SES for the analysis: income, asset, expenditure, living condition, housing condition and education. We find a greater probability of care provision to parents among those in higher SES categories for some SES measures, compared to the lowest category. However, after considering the survival probability of parents, the relationship is reversed and the probability of care provision is found to be greater among lower SES individuals. The association is more pronounced among males. The association is likely to be partly mediated by care needs of parents. These results suggest a higher burden of care disproportionately falls in low SES individuals.

